# A nomogram based on combining systemic and hepatic inflammation markers for predicting microscopic bile duct tumour thrombus in hepatocellular carcinoma

**DOI:** 10.1186/s12885-021-07956-9

**Published:** 2021-03-12

**Authors:** Jun-Yi Wu, Ju-Xian Sun, Jia-Yi Wu, Xiao-Xiao Huang, Yan-Nan Bai, Yong-Yi Zeng, Zhi-Bo Zhang, Shu-Qun Cheng, Mao-Lin Yan

**Affiliations:** 1 Department of Hepatobiliary Surgery, Fujian Provincial Hospital, the Shengli Clinical Medical College of Fujian Medical University, Fuzhou, 350001 Fujian China; 2 Department of Hepatic Surgery VI, The Eastern Hepatobiliary Surgery Hospital, Second Military Medical University, 225 Changhai Road, Shanghai, 200433 China; 3 Department of Hepatobiliary Surgery, Mengchao Hepotabiliary Hospital of Fujian Medical University, Fuzhou, China; 4 Department of Hepatobiliary Surgery, The First Affiliated Hospital of Fujian Medical University, Fuzhou, China

**Keywords:** Hepatocellular carcinoma, Bile duct tumour thrombus, Inflammation, Predicting micro-BDTT

## Abstract

**Background:**

Bile duct invasion is a relatively rare event and is not well characterised in hepatocellular carcinoma (HCC). It remains very difficult to diagnose HCC with bile duct tumour thrombus (BDTT) before surgery. Increasing evidence has revealed that inflammation plays a critical role in tumorigenesis. This study aimed to develop nomograms based on systemic and hepatic inflammation markers to predict microscopic BDTT (micro-BDTT) before surgery in HCC.

**Methods:**

A total of 723 HCC patients who underwent hepatectomy as initial therapy between January 2012 and June 2020 were included in the study. Logistic regression analysis was used to identify independent risk factors for micro-BDTT. The nomograms were constructed using significant predictors, including α-fetoprotein (AFP), alkaline phosphatase (ALP), direct bilirubin (DB), prognostic nutritional index (PNI), and γ-glutamyl transferase (γ-GT)/alanine aminotransferase (ALT). The prediction accuracies of the nomograms were evaluated using the area under the receiver operating characteristic (ROC) curve.

**Results:**

AFP, ALP, DB, PNI, and γ-GT/ALT were independent risk factors for predicting micro-BDTT (*P* = 0.036, *P* = 0.004, *P* = 0.013, *P* = 0.012, and *P* = 0.006, respectively), which were assembled into the nomograms. The area under the ROC curve of the nomograms combining PNI and γ-GT/ALT for predicting micro-BDTT was 0.804 (95% confidence interval [CI]: 0.730–0.878). The sensitivity and specificity values when used in predicting micro-BDTT before surgery were 0.739 (95% CI: 0.612–0.866) and 0.781 (95% CI: 0.750–0.813), respectively.

**Conclusions:**

The nomogram based on combining systemic and hepatic inflammation markers is suitable for predicting micro-BDTT before surgery in HCC patients, leading to a rational therapeutic choice for HCC.

## Background

Bile duct tumour thrombus (BDTT) is a relatively rare event but a well-known presentation in hepatocellular carcinoma (HCC) with a reported incidence of 1.2–12.9% [1–3]. The first description of BDTT was reported in 1947, which always presented with obstructive jaundice [4]. HCC with BDTT has unique clinicopathological features, such as poor differentiation, an infiltrative pattern, and a high incidence of vascular invasion [5–7]. BDTT has been classified into two types: macroscopic BDTT, which indicates that invasion of the tumour thrombus was in the first branches of the bile duct and the common hepatic duct, and microscopic BDTT, which indicates that invasion of tumour thrombus was in the second and more peripheral branches of the bile duct [8]. Because of its rare incidence, the prognostic impact of BDTT is still controversial. However, there is a general consensus that HCC patients with BDTT have poorer prognoses than those without BDTT [8–10]. In addition, patients with HCC and BDTT had a higher propensity for early recurrence [10, 11]. The current diagnostic methods to determine bile duct invasion before surgery are ultrasonic diagnosis, computerised tomography (CT), magnetic resonance imaging (MRI), magnetic resonance cholangiography (MRCP), or endoscopic retrograde cholangiopancreatography (ERCP) [12–14]. Because of advances in imaging an increased understanding of this entity, more and more HCC patients with macroscopic BDTT are being confirmed preoperatively. However, the rate of misdiagnosis of HCC with BDTT before surgery remains very high [15]. In particular, many HCC patients with micro-BDTT cannot be accurately diagnosed preoperatively before surgery. In order to resolve this problem, new markers, including some stem cell markers and small molecule metabolite biomarkers, have been used for diagnosing HCC [15, 16]. However, there is still an urgent requirement for a new diagnostic method that is more accurate and less destructive.

Of note, BDTT, which affects bile drainage, causes biliary obstruction and adversely affects liver function. HCC with BDTT always has reversible hyperbilirubinemia and hypoalbuminemia, which may be caused by a systemic inflammatory response [17]. Therefore, it seems a reasonable approach to predict micro-BDTT with systemic and hepatic inflammation markers. It has been confirmed that tumour growth, development, metastasis, and prognosis are associated not only with tumour characteristics but also with the inflammatory response, which consists of systemic alterations and hepatic inflammation [18–21]. In the clinical setting, C-reactive protein (CRP) is an important systemic inflammation marker that has been confirmed as an independent prognostic indicator of HCC patients [22]. In addition, a series of systemic inflammation markers, including neutrophil-to-lymphocyte ratio (NLR), lymphocyte-to-monocyte ratio (LMR), platelet-to-lymphocyte ratio (PLR), prognostic nutritional index (PNI), and systemic immune-inflammation index (SII), have been scrutinised for the prognosis of tumours, including HCC [23, 24]. These systemic inflammation makers could also predict tumour grade and micro-vascular invasion (MVI) [25]. Based on the inflammatory biomarkers, Li et al. developed nomograms for predicting tumour grade and MVI with high accuracy [23]. Moreover, α-fetoprotein (AFP), γ-glutamyl transferase (γ-GT), and γ-glutamyl transferase to alanine aminotransferase (γ-GT/ALT) reflect hepatic inflammation markers in the liver, which may be independent risk factors for poor prognosis of HCC patients after liver resection [25]. It has been reported that γ-GT/ALT is an independent risk factor for the prognosis of HCC patients after liver resection [26]. At present, based on inflammation markers, many efforts on the preoperative estimation of tumour prognosis, MVI, or tumour grade have been made over the past decade. However, until now, there have been no efforts on the preoperative estimation of micro-BDTT.

We hypothesised that the combination of systemic and hepatic inflammation markers would improve the prediction of HCC with micro-BDTT before surgery.

## Methods

In the present study, we aimed to explore the predictive value of the combination of these haematological parameters in the prediction of HCC with micro-BDTT and to develop nomograms for predicting micro-BDTT before surgery in HCC using only preoperative clinical parameters.

### Patient population

**Fig. 1:**
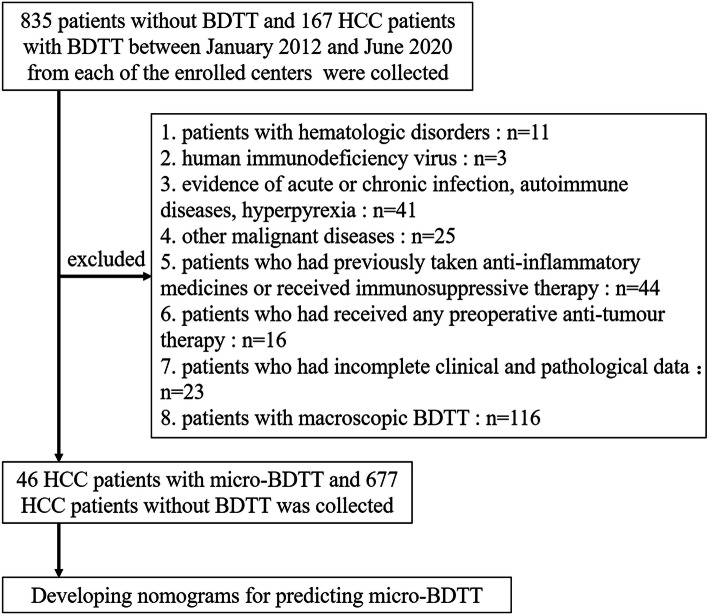
Flow diagram of the study selection process

Patients with HCC between January 2012 and June 2020 from the Fujian Provincial Hospital, Eastern Hepatobiliary Surgery Hospital, The First Affiliated Hospital of Fujian Medical University, and Mengchao Hepotabiliary Hospital of Fujian Medical University were retrospectively reviewed. HCC patients were diagnosed with pathologically confirmed BDTT by two experienced pathologists. The incidence of Micro-BDTT patients was 1.257%. Of these patients, 46 with micro-BDTT were included in our study. The definition of micro-BDTT was in line with a previous report [8]. A total of 835 HCC patients without BDTT were randomly collected from the enrolled centres. Of these patients, 677 HCC patients without BDTT were included for constructing nomograms for predicting micro-BDTT (Fig. 1). Inclusion criteria for this study were: (1) underwent surgical resection; (2) pathological diagnosis of HCC; (3) absence of distant metastasis; and (4) no anticancer treatment for HCC before surgery. The exclusion criteria were: (1) patients with hematologic disorders: chronic leukaemia, myelosuppression, haemophilia, etc.; (2) human immunodeficiency virus, evidence of acute or chronic infection, autoimmune diseases, hyperpyrexia, and patients who had previously taken anti-inflammatory medicines or received immunosuppressive therapy; (3) other malignant diseases: synchronous malignancy or a history of lung cancer, colorectal cancer, gastric cancer, oesophageal cancer, lymphoma, etc.; (4) patients who had received any preoperative anti-tumour therapy; (5) patients who had incomplete clinical and pathological data; and (6) to make a nomogram for predicting with micro-BDTT, patients with macroscopic BDTT, which indicates that invasion of tumour thrombus was in the first branches of the bile duct and the common hepatic duct, were excluded. The study was approved by the Research Ethics Committee of each institution.

### Clinicopathological variables

Preoperative parameters, such as age, sex, serum AFP, ALT, albumin (ALB), γ-GT, alkaline phosphatase (ALP), direct bilirubin (DB), total bilirubin (TB), neutrophil, lymphocyte, monocyte, platelet, maximum size of tumour, number of tumour nodules, and micro-BDTT were collected. The maximum size of the tumour and number of tumour nodules were assessed by preoperative imaging studies. The NLR, PLR, LMR, and PNI were calculated using the following formula: NLR = neutrophil count/lymphocyte count, PLR = platelet count/lymphocyte count, LMR = lymphocyte count/monocyte count, and PNI = 10 × serum albumin (g/dL) + 0.005 × total lymphocyte count (per mm^3^).

### Statistical analysis

Statistical evaluation was conducted using SPSS22.0 (IBM SPSS Inc., Chicago, IL, USA) and R3.1.2 software (Institute for Statistics and Mathematics, Vienna, Austria). Continuous data are expressed as mean ± standard deviation. Receiver operating characteristic (ROC) curve analysis was used to determine the optimal cut-off values for NLR, PLR, LMR, PNI, and γ-GT/ALT based on the maximum Youden’s index (sensitivity + specificity – 1). These cut-off values were used to categorise the high and low groups. Univariate and multivariate logistic regression analyses were used to determine predictive factors for HCC with micro-BDTT, and *P* < 0.05 was considered statistically significant. Confidence intervals (CI) were shown at the 95% confidence level. Based on the results from the multivariate logistic regression analysis, a predictive nomogram was built using R3.1.2 software, which underwent internal validation using the bootstrap method. AUC was calculated to evaluate the performance of the predictive model. A nomogram was developed using the rms package. The nomogram underwent internal validation using the bootstrap method. Each time we do a random sampling with replacement of the same sample size (723), and use these samples to run logistic regression / build nomogram. Sensitivity, specificity, and area under the ROC curve of each nomogram were calculated. These procedures were repeated for 10,000 times. And those 95% CI of sensitivity, specificity, and area under the ROC curve were calculated with statistics, which were obtained from the 10,000 repeated times. The calibration of the nomogram was performed by comparing the predicted and actual probabilities after bias correction.

## Results

### Clinicopathological characteristics

The baseline characteristics of the patients are shown in Table 1. A total of 723 patients with HCC were collected for our analysis, including 677 HCC patients without BDTT and 46 with micro-BDTT. The mean values of WBC, neutrophil, lymphocyte, monocyte, platelet, NLR, PLR, LMR, PNI, and γ-GT/ALT are shown in Table 1. In this study, the upper normal limits for serum ALB, ALT, ALP, γ-GT, and DB values were 40 U/L, 50 U/L, 125 U/L, 60 U/L, and 8 μmol/L, respectively, which were taken as the cut-off values. The two groups differed significantly in ALB (*P* = 0.005), ALP (*P* = 0.000), γ-GT (P = 0.000), DB (P = 0.000), AFP (*P* = 0.002), and γ-GT/ALT (P = 0.000). HCC patients with micro-BDTT had a higher level of ALP, γ-GT, DB, and γ-GT/ALT and a lower level of ALB than those in HCC patients without BDTT. There were no significant differences in age, sex, HBsAg, tumour diameter, tumour number, WBC count, neutrophil count, lymphocyte count, platelet count, NLR, PLR, LMR, and PNI between the groups.

**Table 1:** Clinicopathological features

Variable	without BDTT (*n* = 677)	with micro-BDTT (*n* = 46)	*P*-value
Age (years)			0.552
> 60	220	33	
≤60	457	13	
Sex			0.483
Male	571	37	
Female	106	9	
WBC (*10^9^/ L)	6.17 ± 2.39	5.92 ± 2.52	0.181
Neutrophil (*10^9^/L)	3.87 ± 2.15	3.62 ± 1.93	0.961
Lymphocyte (*10^9^/ L)	1.68 ± 0.69	1.56 ± 0.70	0.872
Monocyte (*10^9^/ L)	0.48 ± 0.24	0.42 ± 0.22	0.594
Platelets (*10^9^/ L)	177.31 ± 75.69	175.59 ± 87.49	0.668
ALB (U/L)			
> 40	498	25	0.005
≤ 40	179	21	
ALP (U/L)			0.000
> 125	138	26	
≤ 125	539	20	
γ-GT (U/L)			0.000
> 60	357	38	
≤ 60	320	8	
DB (μmol/L)			0.000
> 8	130	22	
≤ 8	547	24	
ALT (U/L)			0.290
> 50	205	18	
≤ 50	472	28	
HbsAg			0.916
+ -	570	39	
-	107	7	
γ-GT/ALT	2.53 ± 2.86	6.29 ± 5.28	0.000
NLR	2.79 ± 2.87	2.58 ± 1.53	0.339
PLR	116.48 ± 62.42	119.62 ± 48.34	0.222
LMR	4.10 ± 2.64	4.56 ± 2.74	0.146
PNI	50.99 ± 6.27	46.70 ± 7.80	0.178
Child-Pugh			0.465
A	647	45	
B-C	30	1	
AFP (μg/L)			0.002
≥ 400	244	27	
< 400	433	19	
Tumor size (cm)			0.401
> 5	278	30	
≤ 5	399	16	
Tumor number (n, %)			0.079
1	467	26	
> 1	210	20	

### Prognostic value of systemic and hepatic inflammation markers

The results of the univariate analysis for micro-BDTT are presented in Table 2. Univariate analysis showed that ALB, ALP, γ-GT, DB, AFP, γ-GT/ALT, and PNI were significantly associated with HCC with micro-BDTT. Multivariate regression performed on these significant factors showed that ALP (OR: 2.780, 95% CI: 1.395–5.532, *P* = 0.004), DB (OR: 2.367, 95% CI: 1.199–4.673, *P* = 0.013), AFP (OR: 2.027, 95% CI: 1.047–3.924, *P* = 0.036), PNI (OR: 0.934, 95% CI: 0.885–0.985, P = 0.013), and γ-GT/ALT (OR: 1.115, 95% CI: 1.032–1.205, *P* = 0.006), as shown in Table 3, which were independently associated with HCC with micro-BDTT.

**Table 2:** Univariate logistic regression analysis of micro-BDTT presence based on preoperative data

Variable		Micro-BDTT	
	OR	95%CI	*P*
Age (> 60 VS ≤ 60 years)	0.818	0.422–1.586	0.553
Sex (Male VS Female)	0.763	0.358–1.628	0.484
WBC (*10^9^/L)	0.951	0.831–1.088	0.461
Neutrophil (*10^9^/ L)	0.940	0.802–1.102	0.445
Lymphocyte (*10^9^/ L)	0.751	0.465–1.212	0.241
Monocyte (*10^9^/ L)	0.242	0.046–1.274	0.094
Platelets (*10^9^/ L)	1.000	0.996–1.004	0.882
ALB (> 40 VS ≤ 40 U/L)	0.428	0.234–0.783	0.006
ALP (> 125 VS ≤ 125 U/L)	5.078	2.753–9.365	0.000
γ-GT (> 60 VS ≤ 60 U/L)	4.258	1.957–9.262	0.000
DB (> 8 VS ≤ 8 μmol/L)	3.857	2.097–7.094	0.000
ALT (> 50 VS ≤ 50 U/L)	1.480	0.801–2.736	0.211
HbsAg (+ VS -)	1.046	0.456–2.400	0.916
γ-GT/ALT	1.219	1.133–1.313	0.000
NLR	0.967	0.845–1.106	0.621
PLR	1.001	0.996–1.005	0.737
LMR	1.045	0.967–1.129	0.270
PNI	0.904	0.863–0.946	0.000
Child-Pugh (A VS B-C)	2.087	0.278–15.653	0.474
AFP (> 400 VS ≤ 400 μg/L)	2.522	1.374–4.630	0.003
Tumor size (> 5 VS ≤ 5 cm)	1.306	0.699–2.443	0.403
Tumor number (> 1 VS ≤ 1)	1.711	0.934–3.133	0.082

**Table 3:** Multivariate logistic regression analysis of micro-BDTT presence based on preoperative data

Variable		Micro-BDTT	
	OR	95%Cl	*P*
ALB (> 40 VS ≤ 40 U/L)			0.344
ALP (> 125 VS ≤ 125 U/L)	2.780	1.395–5.532	0.004
γ-GT (> 60 VS ≤ 60 U/L)			0.330
DB (> 8 VS ≤ 8 μmol/L)	2.367	1.199–4.673	0.013
γ-GT/ALT	1.115	1.032–1.205	0.006
PNI	0.934	0.885–0.985	0.013
AFP (> 400 VS ≤ 400 μg/L)	2.027	1.047–3.924	0.036

### Development of a predicting nomogram

**Fig. 2:**
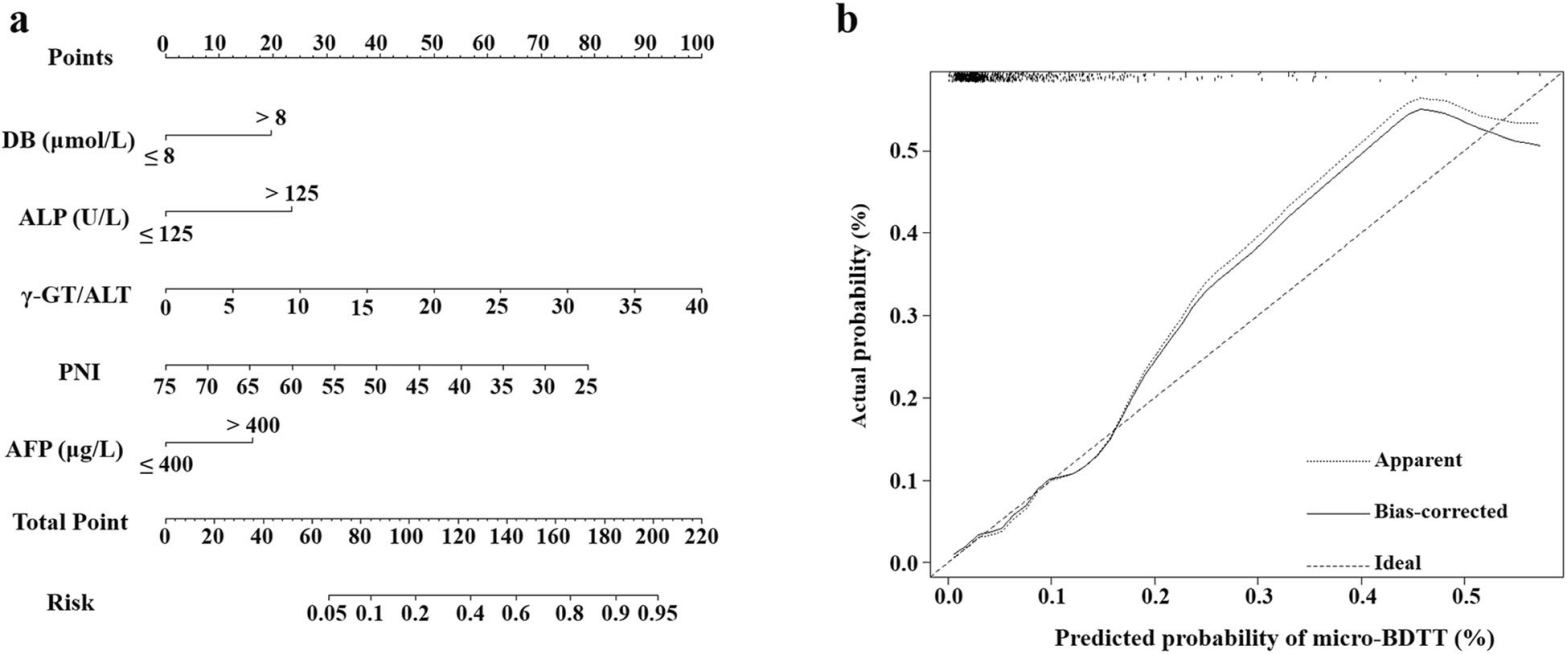
Nomogram to estimate HCC with micro-BDTT presence preoperatively. (a) Nomogram for predicting HCC with micro-BDTT. (b) Calibration plot of the nomogram for predicting the risk of micro-BDTT in HCC

**Fig. 3:**
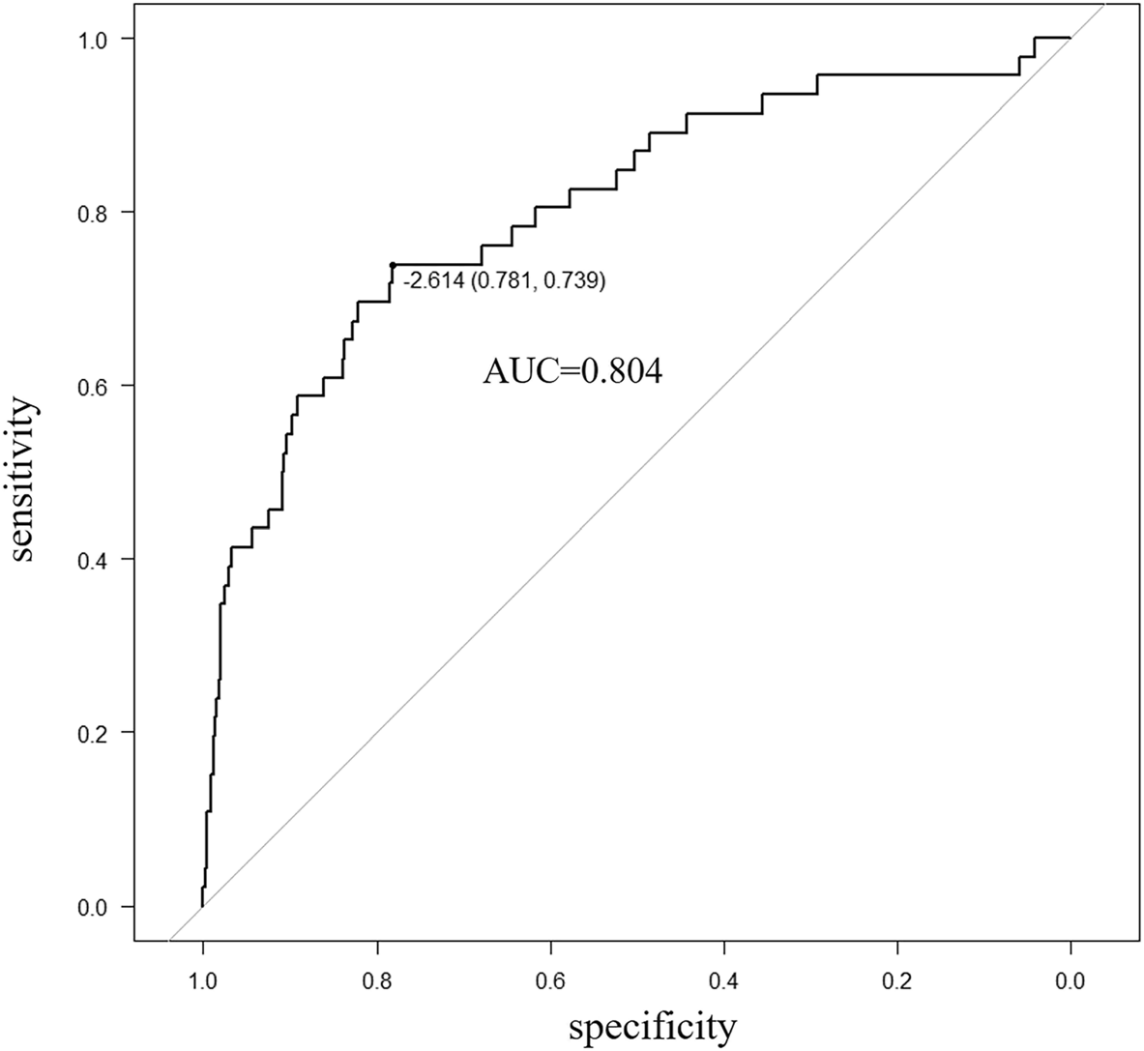
The accuracy of the nomogram for predicting micro-BDTT in HCC using ROC curve

Based on the significant independent variables, a nomogram for predicting the probability of micro-BDTT in HCC was developed. By drawing a straight line after summing up the score assigned to each variable, we could easily obtain the total points, which were converted to predict the probability of HCC with micro-BDTT (Fig. 2a). Patients with a higher total score tended to obtain a higher probability of HCC with micro-BDTT. The accuracy of the micro-BDTT nomogram model was favourable with an area under the ROC curve of 0.804, as shown in Fig. 3. The performance of the nomogram was validated internally with a C-index of 0.804 (95% CI: 0.730–0.878). The calibration curves via internal validation showed good agreement between the predicted and actual probability of micro-BDTT (Fig. 2b). The sensitivity and specificity when used in predicting micro-BDTT before surgery were 0.739 (95% CI: 0.612–0.866) and 0.781 (95% CI: 0.750–0.813), respectively (Table 4).

**Table 4:** Accuracy of the prediction score of the nomogram for estimating the risk of micro-BDTT presence in HCC

Variable	Value (95% CI)
Sensitivity, %	0.739 (0.612–0.866)
Specificity, %	0.781 (0.750–0.813)
Youden Index	0.512
Area under ROC curve	0.804 (0.730–0.878)

## Discussion

HCC with bile duct invasion is vastly rarer than that with vascular invasion. Because of the rare incidence of BDTT, there is insufficient data to systematically analyse the prognostic implications of BDTT. However, many studies support the hypothesis that the prognosis of HCC with BDTT is worse than that of HCC without invasion [8–10]. HCC with BDTT was associated with more advanced-stage HCC with adverse histological features, including higher rates of MVI and poor differentiation [5–7]. Liu et al. also reported that patients with BDTT extending to the common bile duct usually have an unfavourable prognosis, even after aggressive surgery [27]. Although Meng et al. showed that macro-BDTT but not micro-BDTT was an independent risk factor affecting the prognosis of patients with HCC, the number of HCC patients with micro-BDTT (only 7 patients) was too small [28]. More importantly, Kim et al. demonstrated that the prognosis of HCC patients with micro-BDTT was worse than that of those without BDTT [29]. The presence of micro-BDTT should, therefore, be considered an adverse prognostic factor after hepatectomy. Surgical treatment for HCC is considered the most effective approach, including those with BDTT; however, the surgical strategy for HCC with BDTT has not been clearly defined in previous studies [30–32]. However, Kasai et al. had showed that in HCC patients with BDTT alone without MVI, extended hepatectomy provided a better prognosis [31]. In addition, neoadjuvant transcatheter arterial chemoembolization (TACE) reduced the surgical risk of curative liver resection and significantly prolonged median survival in patients with HCC with BDTT [32]. Luo et al. also showed that for HCC patients with BDTT, radical hepatic resection and removal of BDTT, combined with TACE, are the best approach [33]. Peng et al. reported that curative resection for HCC with BDTT can prolong survival [34]. These results indicate that the choice of the most appropriate therapeutic strategy is very important for the prognosis of HCC with BDTT. However, misdiagnosis of BDTT before surgery may lead to inappropriate treatments, resulting in poor patient survival. In our data, only two of 46 HCC patients with micro-BDTT were preoperatively diagnosed.

Previous studies have reported that BDTT could appear even in the early stage of HCC, and can also occur in HCC patients with a tumour diameter of < 3 cm, which indicates that the size of HCC tumours is not correlated with the occurrence of BDTT [27, 35]. In our data, we also found that the size of HCC tumours is not correlated with the occurrence of micro-BDTT. In patients with HCC with BDTT, obstructive jaundice is the main clinical manifestation with a higher preoperative bilirubin level because of biliary tract invasion, and biliary duct dilatation is always observed. These can be used to distinguish HCC from BDTT from those without BDTT. However, these features are also observed in other biliary tract diseases, such as hepatic insufficiency, extrahepatic cholangiocarcinoma, choledochal cyst, and common bile duct stone [15]. In addition, because invasion of the tumour thrombus was in the second and more peripheral branches of the bile duct, serum total bilirubin and DB in HCC with micro-BDTT mostly presents in the normal range, or as slightly higher than normal. In addition, HCC with micro-BDTT has always been ignored in dynamic contrast-enhanced CT or MRI. In our data, all patients with micro-BDTT had no clinical symptoms of jaundice. The DB in HCC with micro-BDTT was only slightly higher than that in HCC without BDTT. Therefore, HCC with micro-BDTT was difficult to diagnose before surgery, since it had no visible symptoms.

There is increasing knowledge that inflammation plays an essential role in tumorigenesis and progression, including HCC. As more than 90% of HCC cases occur with hepatic injury and inflammation, the progression of HCC is an inflammation-related carcinogenesis event [36]. The predictive role of systemic inflammation and hepatic inflammation markers in the prognosis of HCC has received more attention in recent years. Systemic inflammatory indexes such as NLR, PLR, LMR, SII, and PNI are emerging as predictors of prognosis, tumour grade, or MVI in HCC [23]. Herein, we found that pre-treatment PNI levels were significantly associated with micro-BDTT. Through multifactor analysis, pre-treatment PNI level was also an independent predictor of HCC with micro-BDTT. PNI is a marker of albumin and lymphocyte, which has been recognised as an indicator of nutritional and immunological status. Additionally, Chan et al. showed that PNI was an independent prognostic factor for HCC patients [37], and the PNI is a simple and useful systemic inflammation marker for predicting the survival of patients with HCC treated with sorafenib [38]. These results indicate that PNI is feasible as a novel indicator of systemic inflammation. In the present study, PNI was an independent prognostic factor for HCC with micro-BDTT, which was added in the nomogram for predicting micro-BDTT before surgery as a marker of systemic inflammation. However, the specific mechanism of these observations is still unclear.

Liver inflammation plays an important role in the development and prognosis of HCC patients. The clinical indicators of liver inflammation, including ALT, AST, ALP, and γ-GT, were confirmed to be positively related to the recurrence and poor prognosis of HCC patients [39]. The ratio of γ-GT/ALT, another index reflecting liver inflammation, is a powerful prognostic factor for HCC patients. It was found that the ratio of γ-GT/ALT is a convenient prognostic marker for HCC after hepatic resection [25]. In our data, we found that DB, ALP, and γ-GT/ALT were significant prognostic factors of micro-BDTT in patients with HCC. However, the exact mechanisms underlying these observations are still unclear. AFP was also identified as an independent predictor of HCC with micro-BDTT. In light of these findings, we suggest that HCC patients with lower pre-treatment PNI, higher DB, ALP, AFP, and γ-GT /ALT levels may be potential candidates for micro-BDTT before surgery.

We successfully developed a predictive nomogram for predicting micro-BDTT before surgery in HCC by combining systemic and hepatic inflammation markers. We generalised the sensitivity, specificity, positive predictive value, and negative predictive value in estimating the risk of micro-BDTT. Patients with a high score have a high risk of micro-BDTT. As inflammation-based indexes are routinely available and can be measured accurately, the establishment of a predictive model based on inflammation-based indexes may become a useful and inexpensive approach to predict micro-BDTT before surgery. Furthermore, it can provide guidance for choosing more suitable therapeutic strategies for patients.

There are undoubtedly some limitations to our investigation. First, selection bias could have been present because HCC patients without micro-BDTT did not include all HCC patients without micro-BDTT from multi-centres. Second, the number of patients with micro-BDTT was fairly small. However, because of the low incidence of micro-BDTT, the number of cases is difficult to increase. We had already collected HCC patients with micro-BDTT from four centres. Only 46 patients with micro-BDTT were included in our study. Third, since this was a retrospective study, CRP was not routinely measured during the study period. Nevertheless, CRP was detected in only 21 (5.02%) patients in our study. More research is warranted concerning the relationship between micro-BDTT and CRP. In addition, it is necessary to improve the nomogram with more factors. Specific markers, including some stem cell markers and small molecule metabolite biomarkers, may also work as good non-invasive biomarkers for predicting HCC with micro-BDTT. Fourth, further external validation is needed to confirm the reliability of our predictive model through an independent and larger dataset. To our knowledge, this is the first study to construct a predictive nomogram including pre-treatment risk factors for predicting HCC patients with micro-BDTT; however, there are still many deficiencies. Hence, further large-scale, prospective, and multi-centre studies are needed to confirm the results.

## Conclusions

In conclusion, our study highlighted the importance of systemic inflammation and hepatic inflammation in predicting HCC with micro-BDTT. The novel inflammation-based model provided an optimal preoperative estimation of HCC with micro-BDTT, which could help choose suitable surgical methods for HCC with micro-BDTT. Nevertheless, further studies are needed to verify the effectiveness and practicability of the nomograms.

## Data Availability

The datasets used and analyzed in our study are available from the corresponding authors upon reasonable request.

## Abbreviations

BDTT: Bile duct tumour thrombus
HCC: hepatocellular carcinoma
CT: computerised tomography
MRI: magnetic resonance imaging
MRCP: magnetic resonance cholangiography
ERCP: endoscopic retrograde cholangiopancreatography
CRP: C-reactive protein
NLR: neutrophil-to-lymphocyte ratio
LMR: lymphocyte-to-monocyte ratio
PLR: platelet-to-lymphocyte ratio
PNI: prognostic nutritional index
SII: systemic immune-inflammation index
MVI: micro-vascular invasion
AFP: α-fetoprotein
γ-GT: γ-glutamyl transferase
γ-GT/ALT: γ-glutamyl transferase to alanine aminotransferase
ALP: alkaline phosphatase
DB: direct bilirubin
ALB: albumin
